# The relationship between self-efficacy, health literacy, and quality of life in patients with chronic diseases: a cross-sectional study in China

**DOI:** 10.3389/fpubh.2024.1430202

**Published:** 2024-09-19

**Authors:** Ying’e Gao, Yujia Zheng, Yuanyuan He, Jingjing Lin, Fangyi Liu, Jie Fu, Rongjin Lin

**Affiliations:** ^1^School of Nursing, Fujian Medical University, Fuzhou, China; ^2^School of Basic Medical Sciences, Fujian Medical University, Fuzhou, China; ^3^The First Affiliated Hospital of Fujian Medical University, Fuzhou, China

**Keywords:** health literacy, self-efficacy, quality of life, chronic disease, four-way decomposition

## Abstract

**Background:**

Self-efficacy and health literacy are closely related to the quality of life in patients with chronic diseases; however, it remains unclear whether their combined effects on the quality of life (QoL) in these patients operate through mediation, interaction, or a combination of both.

**Methods:**

The research occurred in China between July 10 and September 15, 2021. A multi-stage random sampling technique was utilized to gather information on self-efficacy, health literacy, and QoL among individuals with chronic diseases. Linear regression models investigated the relationships between these patients’ self-efficacy, health literacy, and QoL. Additionally, the four-way decomposition method was used to decompose the overall effects of self-efficacy and health literacy on the QoL in patients with chronic diseases.

**Results:**

Significant correlations were found between self-efficacy, health literacy, and QoL among individuals with chronic diseases (all *p* < 0.05). In the four-way decomposition results, the results of the European Quality of Life Five Dimension Five Level (EQ-5D-5L) displayed the interaction effects mediated by self-efficacy, and the reference interaction effects were not significant, with small effect sizes observed. The influence of health literacy levels on the QoL in these patients was primarily attributed to the controlled direct effect (CDE), accounting for approximately 86.12% [excess relative risk = 0.00415; 95% CI: 0.00326, 0.00504; *p* < 0.0001]. The proportion solely attributable to the pure indirect effect (PIE) of self-efficacy was 14.5% [excess relative risk = 0.0007; 95% CI: 0.00031, 0.00109; *p* < 0.0001]. In the EQ visual analog scale (EQ-VAS) results, the proportion of the controlled direct effect was 84.9% [excess relative risk = 0.62443; 95% CI: 0.52269, 0.72618; *p* < 0.0001], while the proportion solely attributable to the pure indirect effect of mediation was 14.8% [excess relative risk = 0.10876; 95% CI: 0.06409, 0.15344; *p* < 0.0001].

**Conclusion:**

Self-efficacy and health literacy primarily influence QoL in patients with chronic diseases through controlled and pure indirect effects. Enhancing patients’ health literacy and self-efficacy can contribute to improving their QoL.

## 1 Introduction

Chronic non-communicable diseases (NCDs), abbreviated as chronic diseases, are complex conditions caused by various factors, including physiological abnormalities, genetic predisposition, environmental influences, and personal behaviors, rendering them particularly challenging to address comprehensively. Common NCDs encompass cardiovascular diseases (such as heart disease and stroke), chronic respiratory diseases (like chronic obstructive pulmonary disease), diabetes (type 1 and type 2), and cancers (a variety of diseases characterized by abnormal cell growth) (1). The global impact of non-communicable diseases is staggering, with approximately 74% of global deaths attributed to NCDs each year (1). It is projected that by 2030, NCDs will surpass infectious diseases to become the leading cause of global mortality. In China, the burden of NCDs has dramatically increased over the past 20 years, manifesting as continuous rises in incidence, disability rates, and mortality rates. The National Health Services Survey Report on the Sixth National Health Services Statistics shows that major NCDs such as cardiovascular diseases, diabetes, and cancer account for over 90% of China’s disease-related economic burden. The prevalence of NCDs among the Chinese population aged 55 to 64 is 48.4%, while it reaches 62.3% among those aged 65 and above (2). In May 2022, during the 75th World Health Assembly, the World Health Organization (WHO) announced ambitious goals for preventing and controlling NCDs. These objectives aim to inspire international action and mobilize resources to alleviate the impact of non-communicable diseases on global health and well-being (3).

Quality of Life (QoL) is a complex concept that incorporates an individual’s holistic perception of their living conditions, comprising aspects such as physical health, mental well-being, social connections, and personal satisfaction. It is a subjective assessment of one’s satisfaction and contentment with one’s life, considering one’s desires, values, and cultural background (4, 5). NCDs are characterized by insidious onset, long disease duration, and disease recurrence. The long-term recurrence and continuous drug treatment of NCDs not only significantly impact patients’ physical and mental well-being but also elevate the caregiving responsibilities of patients, their families, and society at large. Faced with incurable NCDs, patients May experience feelings of powerlessness and negativity, leading to reduced treatment compliance and effectiveness, thereby seriously compromising their daily life quality. International research has demonstrated that chronic conditions such as stroke (6) and hypertension (7) adversely affect patients’ physiological function and mental health. Martins et al. (8) found that NCDs patients with sleep disorders exhibited issues such as daytime sleepiness, decreased physical health, and an increased risk of cognitive impairment, with their QoL being significantly lower than that of healthy older adults. In the United States, cardiovascular diseases, cancer, chronic respiratory diseases, and diabetes—referred to as the “Big Four” non-communicable diseases—are prominent factors influencing both morbidity and mortality rates. These conditions pose significant health risks and profoundly impact patients’ ability to manage their daily lives. The complex treatment regimens, potential complications, and lifestyle changes associated with these diseases can severely impair patients’ self-care abilities and overall quality of life. This underscores the critical necessity for implementing comprehensive strategies to address the multifaceted challenges of non-communicable diseases (9).

Health literacy entails obtaining, comprehending, assessing, and utilizing health-related information and services to make informed decisions that promote personal health, incorporating cognitive abilities and social skills (10). Existing studies (11–14) indicate that individuals with lower health literacy often experience poorer health outcomes, including increased healthcare expenditures, higher hospitalization, and mortality rates. Approximately 39% of the global population is estimated to have insufficient health literacy (15), with only 12% possessing high health literacy levels in the United States, 47.6% in Europe, and over half in Canada (16–18). The 2021 China Health Literacy Survey reveals that a quarter of Chinese residents, totaling 25.40%, demonstrate health literacy (19), significantly lower than that of other countries. The importance of health literacy becomes even more pronounced in the context of chronic disease management. NCDs require ongoing self-management and adherence to complex treatment regimens, underscoring the pivotal role of health literacy in ensuring effective disease management and enhancing the quality of life for patients. Elevated health literacy enables individuals to comprehend their conditions, interact proficiently with healthcare professionals, and make informed choices regarding their health. Consequently, higher levels of health literacy May substantially improve health outcomes and enrich the quality of life for individuals grappling with NCDs.

Self-efficacy, a pivotal positive psychological construct, denotes an individual’s confidence in their capacity to accomplish specific behavioral objectives within a given domain (20). This construct is considered instrumental in enhancing the life quality of individuals afflicted with chronic conditions (21). Empirical evidence suggests that individuals with rheumatoid arthritis who possess elevated levels of self-efficacy are less susceptible to psychological distress, thereby correlating with an enhanced life quality (22). Wang et al. demonstrated that targeted interventions aimed at bolstering self-efficacy in individuals with hepatitis B can markedly elevate their life quality (21). Furthermore, Tattersall et al. emphasized the imperative of augmenting patients’ confidence and self-efficacy, thereby promoting autonomous decision-making and the application of personal knowledge and skills in the management of NCDs (23).

Given the large patient population, high mortality rates, and heavy disease burden associated with NCDs, improving patient QoL has become a significant issue for scholars and a subject worthy of perpetual exploration (24). Research has established a correlation between health literacy, self-efficacy, and the life quality of those with chronic conditions (25). Usser et al. have delineated a link between health literacy and self-efficacy, noting that individuals with greater health literacy exhibit higher levels of self-efficacy (26). This self-efficacy can direct health behaviors, potentially managing or decelerating the emergence or progression of diseases, thereby indirectly ameliorating the life quality of patients (20). However, these studies have yet to consider multiple factors simultaneously, and each factor’s synergistic effects, magnitudes, and underlying mechanisms on the QoL still need to be clarified.

The purpose of this nationally representative survey is to investigate the correlation between health literacy, self-efficacy, and the QoL among patients with NCDs. This study aims to establish a theoretical foundation for enhancing the well-being of NCDs patients in China.

## 2 Methods

### 2.1 Study design and participants

This study collected data from a cross-sectional survey conducted nationwide in China from July 10 to September 15, 2021. The survey covered 120 cities across 23 provinces in mainland China and utilized data from the “Seventh National Population Census of 2021.” Stratified random sampling was employed based on age, gender, and urban–rural distribution to select the target population. The survey team was comprised of publicly recruited and trained investigators or survey groups with no more than 10 members. Each investigator collected 30 to 90 questionnaires, while each group handled 100 to 200 questionnaires. Questionnaires were distributed face-to-face to residents using the Wen Juan Xing platform (https://www.wjx.cn/).A total of 11,031 individuals participated and completed the electronic questionnaire. The primary subjects of this study were patients with NCDs in China. Participants who responded affirmatively to question 29, “Have you ever been diagnosed by a doctor with any of the following NCDs?” were included in the study, excluding those who selected “None.” The NCDs referenced in question 29 encompassed fractures, cataracts, osteoporosis, arthritis, and other ailments. Two researchers performed consecutive logic checks and questionnaire screenings according to the predetermined screening criteria. Questionnaires failing to meet the following criteria were excluded (1): completion time less than 240 s (2); logical errors in responses (3); incomplete questionnaires (4); duplicate questionnaires (5); identical responses to reverse questions in scales. Ultimately, 2,025 questionnaires were confirmed valid, with male participants accounting for 52.4% (1,061 individuals).

### 2.2 Health literacy

This study utilized the Health Literacy Scale Short Form (HLS-SF12), developed by Duong TV et al. in 2019 (25), to assess health literacy. The HLS-SF12 encompasses three domains: healthcare, disease prevention, and health promotion, and evaluates participants’ health literacy levels through self-reporting. The scale employs a Likert scale (1 = very difficult, 2 = difficult, 3 = easy, 4 = very easy) to measure the perceived difficulty of each item. Scores on the HLS-SF12 range from 12 to 48, where higher scores denote higher health literacy levels. In this study, the Cronbach’s alpha coefficient for the scale was computed as 0.937, indicating strong reliability and validity (26).

### 2.3 Self–efficacy

This study utilized the New General Self-Efficacy Scale (NGSES), developed by Chen et al., to measure patients’ self-efficacy (27). The scale comprises eight items, with respondents rating each item on a five-point Likert scale ranging from 1 (strongly disagree) to 5 (strongly agree). The total score ranges from 8 to 40, with higher scores indicating stronger self-efficacy among patients. In this study, the Cronbach’s alpha coefficient for the scale was calculated to be 0.904, indicating high reliability.

### 2.4 Quality of life

This study utilized the European Quality of Life Five Dimension Five Level (EQ-5D-5L), developed by the Euro Qol Group (27), to evaluate the participants’ quality of life. The EQ-5D-5L comprises a concise self-report system questionnaire (EQ-5D) and a visual analog scale (EQ-VAS). The scale assesses five dimensions: mobility (MO), self-care (SC), usual activities (UA), pain/discomfort (PD), and anxiety/depression (AD). Respondents rated their health issues on a five-point scale within each dimension: “no problems (1)”, “slight problems (2)”, “moderate problems (3)”, “severe problems (4)”, and “extreme problems (5)” (28, 29). A composite numerical score was generated by aggregating the values across these five dimensions to depict the respondent’s health status, with “11111” and “55555” denoting the “best health state” and “worst health state, “respectively. The EQ-5D-5L values tailored for the Chinese population were employed for statistical analysis to translate the EQ-5D states into corresponding scores[^34^]. Furthermore, the EQ-VAS served as a tool for participants to self-assess their overall health status by selecting a number between 0 and 100 to represent their current health condition (27). The Cronbach’s alpha coefficient of EQ-5D-5L scale is 0.857 (30), this scale has demonstrated sufficient reliability and validity (29, 31).

### 2.5 Covariates

We included the following covariates, which were theoretically associated with participants’ health status: gender (Male/Female), age (Age group), area of residence (Rural/Urban), type of hukou (Agricultural, Non-agricultural), religious belief (Yes/No), education level (No formal education, Junior high school and below, High school, Collage, Master or above), work status (No fixed occupation, Retirement, Students), ethnicity (Han Chinese, Other), marital status (Unmarried, Married, Divorced, Widowed), family type (Core family, Intergenerational family, joint family, Married family, Other families, Single parent families, Trunk family), number of children (Childless, 1, 2, ≥3), monthly income (≤ 3000, 3001–6000, 6001–9000, >9000), type of medical insurance (Commercial health insurance, self-paying, resident medical insurance, employee medical insurance, Public expense), disability (Yes/No), number of chronic diseases (1, 2, ≥3), number of medications taken (No, 1, 2, ≥3), alcohol consumption (Yes/No), Smoking (Never, Quit Smoking, Smoking), and body mass index (BMI).

### 2.6 Statistical analysis

**Figure 1:**
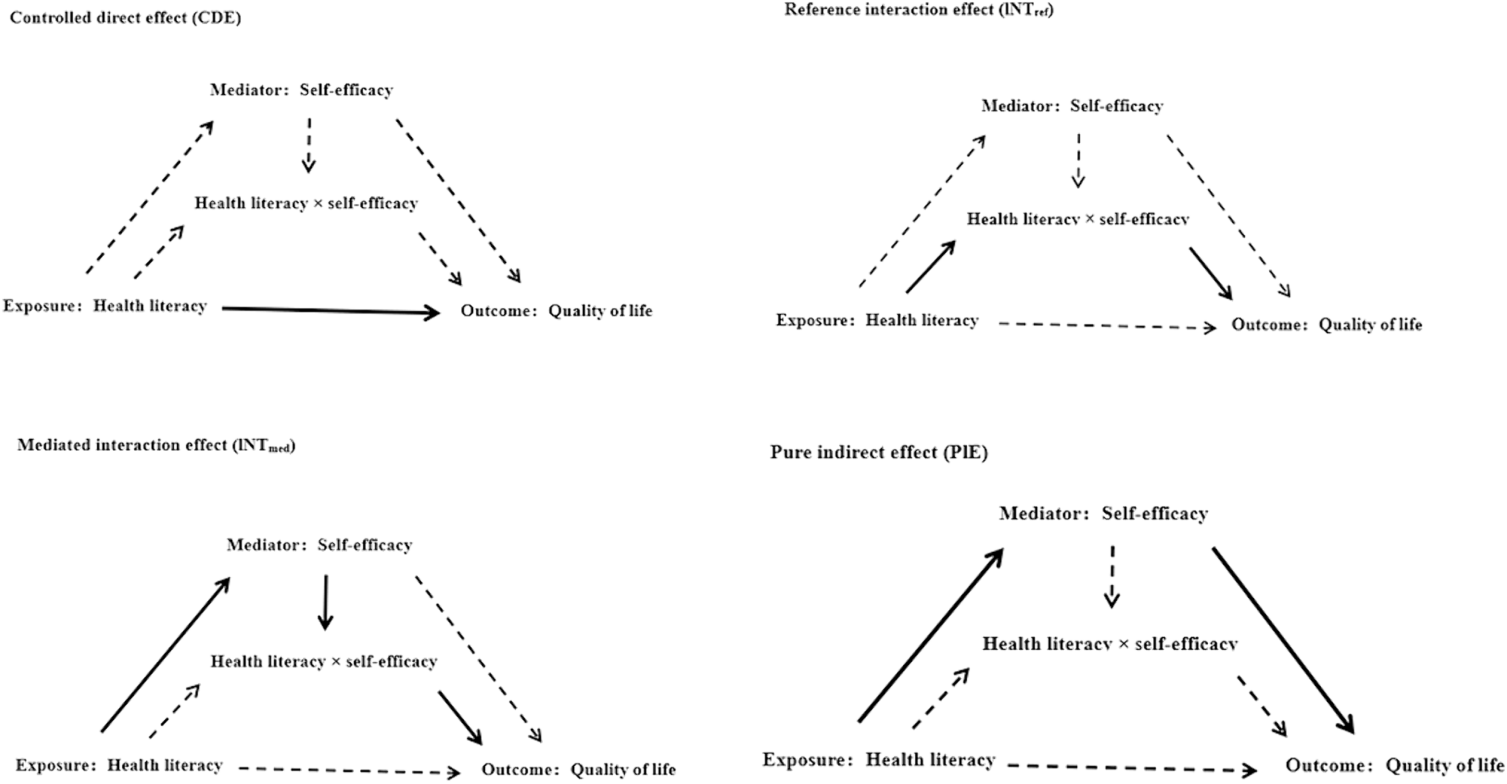
Causal diagram representing 4-way decomposition of the relationship between self-efficacy and health literacy and QoL in patients with chronic diseases.

We performed analyses using Stata 17.0 and R 4.3.3. Categorical variables were expressed as frequencies and percentages (%), while continuous variables were presented as means and standard deviations (SD). Linear regression models were employed to estimate odds ratios with 95% confidence intervals (CI) and to explore the relationships between self-efficacy, health literacy, and QoL in Chinese patients with NCDs. Model 1 remained unadjusted for potential covariates. Model 2 included adjustments for gender, age, ethnicity, family type, area of residence, hukou type, religious belief, monthly income, education level, marital status, number of children, work status, and insurance type. Model 3, an extension of Model 2, further adjusted for patients’ disability status, medication use, Smoking, and alcohol consumption. Additionally, we employed the “med4way” Stata command to conduct a four-way decomposition analysis (32), probing the interactive and mediating impacts of self-efficacy on the correlation between health literacy and QoL in Chinese patients with NCDs. The overall impact of exposure on the outcome was subdivided into four distinct components: (1) Controlled Direct Effect (CDE): the direct impact of health literacy on QoL among chronic patients, without considering the mediating role of self-efficacy and the interaction between health literacy and self-efficacy; (2) Reference Interaction Effect (INTref): the influence of health literacy on patients’ QoL due to the mediating role of self-efficacy and the interaction between health literacy and self-efficacy; (3) Mediated Interaction Effect (INTmed): the effect of health literacy on patients’ QoL mediated by self-efficacy and the interaction between health literacy and self-efficacy; (4) Pure Indirect Effect (PIE): the impact of health literacy on QoL in chronic patients solely mediated by self-efficacy (Figure 1). We utilized 1000 bootstrap resampling iterations to compute bias-corrected 95% confidence intervals. An interval that does not include zero signifies a statistically significant effect.

## 3 Results

### 3.1 Sample characteristics

Table 1 presents the self-reported sociodemographic characteristics of patients with NCDs. A total of 2,025 subjects participated in this study. Patients aged 45–60 and those older than 60 represented a more significant proportion, accounting for 34.5 and 35.2%, respectively. Regarding gender distribution, male patients outnumbered females, constituting 52.4% (1,061 individuals). Among the patients with NCDs, 70.7% (1,431) had a single chronic condition, while 29.3% (594) had two or more comorbidities. Regarding medication, 31.3% (634) of the patients with NCDs had not initiated relevant treatments. Additionally, approximately 33.3% of the respondents reported an average monthly income below RMB 3,000 (approximately US$417.63), and over half of the participants had a monthly income ranging from RMB 3,000 to RMB 9,000 (approximately US$417.63 to US$1,252.89).

**Table 1:** Sample characteristics (*N* = 2025).

Characteristic	Overall (*n* = 2025)	Characteristic	Overall (*n* = 2025)
**Gender**		**Race**	
Female	964 (47.6)	Han Chinese	1900 (93.8)
Male	1061 (52.4)	Other	125 (6.2)
**Age(years)**		**Religious belief**	
≤18	42 (2.1)	No	1944 (96.0)
19–45	571 (28.2)	Yes	81 (4.0)
46–60	699 (34.5)	**Education level**	
>60	713 (35.2)	No formal education	185 (9.1)
**Residence**		Junior high school and below	680 (33.6)
Urban	1431 (70.7)	High school, junior college	647 (32.0)
Rural	594 (29.3)	College	424 (20.9)
**Type family**		Master or above	89 (4.4)
Core family	890 (44.0)	**Marital status**	
Intergenerational family	49 (2.4)	Marred	1581 (78.1)
Joint family	102 (5.0)	Unmarried	224 (11.1)
Married family	367 (18.1)	Divorce	59 (2.9)
Other families	98 (4.8)	Widowed	161 (8.0)
Single parent families	78 (3.9)	**Number children**	
Trunk family	441 (21.8)	Childless	280 (13.8)
**Chronic disease number**		A child	759 (37.5)
1	1431 (70.7)	Two children	650 (32.1)
2	409 (20.2)	≥Three children	336 (16.6)
≥3	185 (9.1)	**Nature account**	
**Monthly income**		Agricultural	840 (41.5)
≤3000	673 (33.2)	Non-agricultural	1185 (58.5)
3001–6000	780 (38.5)	**Career status**	
6001–9000	331 (16.3)	Incumbency	756 (37.3)
>9000	241 (11.9)	No fixed occupation	633 (31.3)
**Health insurance**		Retirement	487 (24.0)
Commercial health insurance	52 (2.6)	Student	149 (7.4)
Employee medical insurance	657 (32.4)	**Disability status**	
Public expense	46 (2.3)	No	1817 (89.7)
Resident medical insurance	988 (48.8)	Yes	208 (10.3)
Self-pay	282 (13.9)	**Smoking status**	
		Never	1328 (65.6)
**Medications are taken**		Quit smoking	327 (16.1)
No	634 (31.3)	Smoking	370 (18.3)
1	542 (26.8)	**Drinking status**	
2	454 (22.4)	No	1100 (54.3)
≥3	395 (19.5)	Yes	925 (45.7)
**BMI**	22.83 (3.46)		
**EQ VAS**	74.93 (18.14)		
**Score EQ**	0.89 (0.16)		
**Score NGSES**	28.29 (5.26)		
**Score HLS-SF12**	31.58 (8.06)		

### 3.2 Descriptive results of self–efficacy, health literacy, and quality of life in patients with chronic diseases

The surveyed participants demonstrated a relatively high level of health literacy, with a mean score of 31.58 (SD = 8.06; range: 12–48). Additionally, participants exhibited high levels of self-efficacy, with a mean score of 28.29 (SD = 5.26; range: 8–40). Regarding QoL indicators, the EQ-5D-5L indicated a high level of QoL among participants, with a mean score of 0.89 (SD = 0.16; range: −0.391-1.000) (Table 1).

### 3.3 The associations between health literacy and quality of life in patients with chronic diseases

The results from the linear regression model examining the association between health literacy and QoL in patients with NCDs are presented in Table 2. The EQ-5D questionnaire results revealed a positive correlation between health literacy and QoL in patients with NCDs in the original model [*β* (95%CI): 0.006 (0.005, 0.007), *p* < 0.0001]. This correlation persisted across the Q1-Q4 quartiles. After adjusting for sociodemographic variables (Model 2) and NCDs and physical conditions (Model 3), the positive relationship between health literacy and QoL remained significant [Model 2: *β* (95%CI): 0.006 (0.005, 0.007), *p* < 0.0001; Model 3: *β* (95%CI): 0.005 (0.004, 0.006), *p* < 0.0001]. In the EQ-VAS visual analog scale results, a positive correlation was observed between health literacy and EQ-VAS values across all three models [Model 1: β (95%CI): 0.835 (0.744, 0.926), *p* < 0.0001; Model 2: *β* (95%CI): 0.799 (0.709, 0.890), *p* < 0.0001; Model 3: *β* (95%CI): 0.735 (0.643, 0.826), *p* < 0.0001]. This correlation remained consistent across the Q1-Q4 quartiles.

**Table 2:** The relationship between self-efficacy, health literacy, and QoL of patients with chronic diseases.

	EQ-5D						EQ-VAS					
	Model 1		Model 2		Model 3		Model 1		Model 2		Model 3	
	95%CI	*p*	95%CI	*p*	95%CI	*p*	95%CI	*p*	95%CI	*p*	95%CI	*p*
**HLS-SF12**												
**Q1**	Ref		Ref		Ref		Ref		Ref		Ref	
**Q2**	0.099 (0.077,0.121)	<0.0001	0.098 (0.076,0.120)	<0.0001	0.08 (0.059,0.101)	<0.0001	11.118 (8.708,13.528)	<0.0001	10.545 (8.154,12.935)	<0.0001	9.384 (7.007,11.761)	<0.0001
**Q3**	0.122 (0.104,0.139)	<0.0001	0.12 (0.103,0.138)	<0.0001	0.097 (0.081,0.114)	<0.0001	11.908 (10.029,13.786)	<0.0001	11.544 (9.677,13.411)	<0.0001	10.253 (8.381,12.125)	<0.0001
**Q4**	0.131 (0.113,0.149)	<0.0001	0.129 (0.110,0.147)	<0.0001	0.105 (0.088,0.123)	<0.0001	18.157 (16.172,20.142)	<0.0001	17.318 (15.340,19.296)	<0.0001	15.984 (13.991,17.977)	<0.0001
**P for trend**		<0.0001		<0.0001		<0.0001		<0.0001		<0.0001		<0.0001
**NGSES**												
**Q1**	Ref		Ref		Ref		Ref		Ref		Ref	
**Q2**	0.030 (0.011,0.049)	0.002	0.030 (0.011,0.049)	0.002	0.024 (0.007,0.042)	0.007	4.192 (2.119,6.265)	<0.0001	4.187 (2.132,6.242)	<0.0001	3.737 (1.729,5.745)	<0.001
**Q3**	0.081 (0.064,0.099)	<0.0001	0.078 (0.060,0.096)	<0.0001	0.062 (0.045,0.079)	<0.0001	10.039 (8.099,11.979)	<0.0001	9.255 (7.328,11.182)	<0.0001	8.536 (6.642,10.430)	<0.0001
**Q4**	0.063 (0.039,0.087)	<0.0001	0.062 (0.038,0.086)	<0.0001	0.057 (0.035,0.079)	<0.0001	13.184 (10.564,15.804)	<0.0001	12.427 (9.832,15.022)	<0.0001	12.14 (9.607,14.673)	<0.0001
**P for trend**		<0.0001		<0.0001		<0.0001		<0.0001		<0.0001		<0.0001

BMI, body mass index.

Model 1 unadjusted for covariates.

Model 2 was adjusted for age, gender, race, residence, type of family, nature account, monthly income, religious belief, education level, marital status, number of children, career status, and health insurance.

Model 3 was adjusted as model 2 plus medications were taken, smoking status, and drinking status.

### 3.4 The associations between self–efficacy and quality of life in patients with chronic diseases

**Figure 2:**
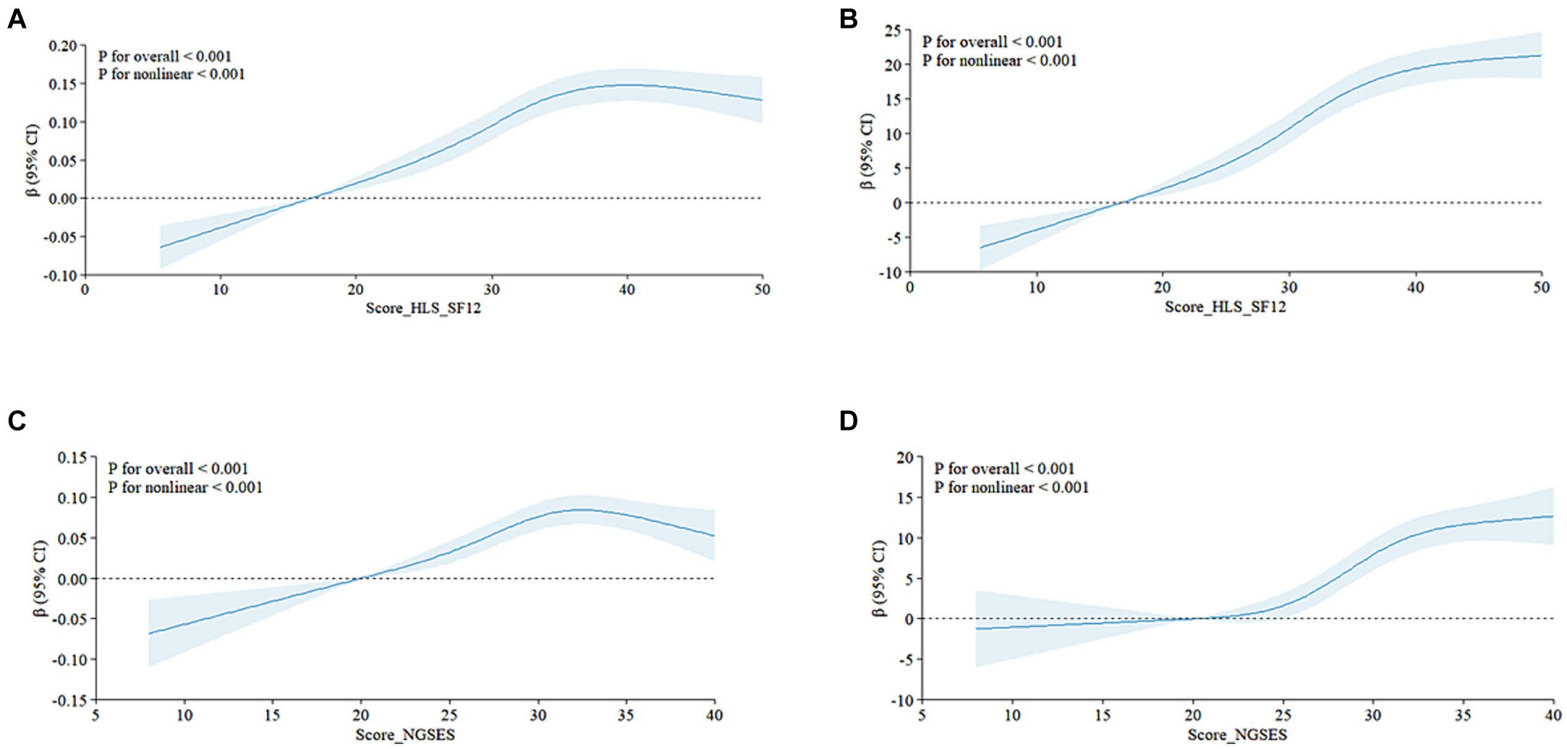
Self-Efficacy and Health Literacy in patients with chronic diseases were nonlinearly associated with QoL The relationship between health literacy and quality of life is shown in figures **(A)** (EQ-5D-5L) and **(B)** (EQ-VAS), and the nonlinear relationship between Self-Efficacy and QoL life is shown in figures **(C)** (EQ-5D) and **(D)** (EQ-VAS). Score HLS-SF12, health literacy score; Score_NGSEES, self-efficacy score; CI, confidence interval.

Table 2 presents the linear regression model examining the relationship between self-efficacy and QoL in patients with NCDs. The EQ-5D questionnaire results indicate a positive correlation between self-efficacy and QoL across the three models [Model1: *β* (95% CI) = 0.006 (0.005, 0.007), *p* < 0.0001; Model2: *β* (95% CI) = 0.006 (0.004, 0.007), *p* < 0.0001; Model3: *β* (95% CI) = 0.005 (0.004, 0.007), *p* < 0.0001]. This correlation persisted across quartiles (Q1-Q4). In the EQ-VAS visual analog scale results, health literacy and EQ-VAS values were positively correlated across the three models [Model1: *β* (95% CI) = 0.880 (0.735, 0.890), *p* < 0.0001; Model2: *β* (95% CI) = 0.814 (0.670, 0.959), *p* < 0.0001; Model3: *β* (95% CI) = 0.778 (0.636, 0.919), *p* < 0.0001]. This correlation was consistently significant across self-efficacy quartiles (Q1-Q4).Additionally, restricted cubic spline (RCS) analysis revealed a significant nonlinear association between self-efficacy and QoL among patients with NCDs (*p* < 0.001, refer to Figures 2A–D). These results indicate a positive correlation between self-efficacy and health literacy with the QoL in patients grappling with NCDs, and this association is statistically significant.

These findings highlight the potential significance of self-efficacy in improving QoL for individuals with NCDs. Therefore, improving patients’ self-efficacy should be a priority in chronic disease management.

### 3.5 Based on the four-way decomposition method, the effects of self-efficacy and health literacy on the quality of life of patients with chronic diseases were analyzed

Table 3 presents the results of the four-way decomposition analysis examining the effects of self-efficacy and health literacy on the QoL in Chinese chronic disease patients. We found that the total effect and pure indirect effect were statistically significant. However, neither the interaction mediated by self-efficacy nor the reference interaction effects were significant, and the effect sizes were small. The results from the EQ-5D questionnaire indicate that approximately 86.12% of the impact of health literacy level on the QoL in chronic disease patients is attributable to direct effects [excess relative risk = 0.00415; 95% CI: 0.00326, 0.00504; *p* < 0.0001]. The proportion of the reference interaction effect (INTref) of self-efficacy between health literacy and QoL is 0.6% [excess relative risk = 0.00003; 95% CI: −0.00002, 0.00008; *p* = 0.197]. Mediated by self-efficacy, the proportion of the reference interaction effect (INTmed) is −1.3% [excess relative risk = −0.00007; 95% CI: −0.00010, −0.00003; *p* < 0.0001], suggesting that the association between health literacy and QoL May be weakened to some extent in patients with NCDs. The proportion of pure indirect effects (PIE) attributed solely to self-efficacy was 14.5% [excess relative risk = 0.0007; 95% CI: 0.00031, 0.00109; p < 0.0001]. In the EQ-VAS visual analog scale results, the proportion of controlled direct effects (CDE) not attributable to mediators or interactions was 84.9% [excess relative risk = 0.62443; 95% CI: 0.52269, 0.72618; *p* < 0.0001]. The proportion of reference interaction effect (INTref) attributable solely to interaction was 0.5% [excess relative risk = 0.00390; 95% CI: −0.00507, 0.1287; *p* = 0.394]. The proportion of mediated interaction effects (INTmed) due to mediators and interaction effects was −0.2% [excess relative risk = −0.00151; 95% CI: −0.00497, 0.00194; *p* = 0.391]. The proportion of pure indirect effects (PIE) attributed solely to intermediaries was 14.8% [excess relative risk = 0.10876; 95% CI: 0.06409, 0.15344; p < 0.0001]. In summary, the total and pure indirect effects were statistically significant. However, both the interaction mediated by self-efficacy and the reference interaction were insignificant and had negligible effect sizes.

**Table 3:** Proportions of the effect of health literacy on QoL of patients with chronic diseases due to mediation and interaction with self-efficacy.

	Score—EQ			EQ-VAS		
	Excess relative risk (95% CI)	P*	Proportion attributable (%)	Excess relative risk (95% CI)	P*	Proportion attributable (%)
CDE	0.00415 (0.00326,0.00504)	<0.0001	86.1	0.62443 (0.52269,0.72618)	<0.0001	84.9
INTref	0.00003 (−0.00002,0.00008)	0.197	0.7	0.00390 (−0.00507,0.1287)	0.394	0.5
INTmed	−0.00007 (−0.00010,−0.00003)	<0.0001	−1.3	−0.00151 (−0.00497,0.00194)	0.391	−0.2
PIE	0.0007 (0.00031,0.00109)	<0.0001	14.5	0.10876 (0.06409,0.15344)	<0.0001	14.8
Total effect	0.00482 (0.00401,0.00562)	<0.0001	100.0	0.73559 (0.64425,0.82693)	<0.0001	100.0

CI, confidence interval; CDE, controlled direct effect; INTmed, mediated interaction; INTref, reference interaction; PIE, pure indirect effect; Total effect = CDE + INTref + INTmed + PIE.

*Adjusted for age, gender, race, residence, type of family, nature account, monthly income, religious belief, education level, marital status, number of children, career status, health insurance, medications taken, smoking status, and drinking status.

These findings shed light on the potential mechanisms through which self-efficacy and health literacy May improve the QoL in patients with NCDs. They also provide empirical evidence supporting the development of targeted intervention measures.

## 4 Discussion

The relationship between health literacy, self-efficacy, and the QoL in patients with NCDs has increasingly garnered research attention. However, the specific mechanisms by which these factors influence QoL still need to be understood, warranting further exploration. Drawing from a large, representative sample of Chinese patients with NCDs, this study arrives at the following conclusions: Utilizing a linear regression model, we found that self-efficacy and health literacy are positively associated with the QoL in patients with NCDs, even after adjusting for confounding factors. Additionally, four-way decomposition analysis indicated that the positive impact of health literacy on QoL predominantly manifests through the control direct effect (CDE), as evidenced by both EQ-5D and EQ-VAS visual scores. Interestingly, self-efficacy did not exhibit a significant interactive mediating effect between health literacy and QoL; its impact was not statistically significant. Moreover, changes in the QoL among patients with NCDs were not influenced by the interaction between health literacy and self-efficacy. As a mediating factor, self-efficacy contributed 14.5 and 14.8% to the total effect on QoL in EQ-5D and EQ-VAS scores, respectively.

These insights offer a novel perspective on self-efficacy and health literacy’s independent and combined roles in influencing QoL among patients with NCDs. They also suggest potential avenues for targeted intervention strategies in the future.

### 4.1 The relationship between the health literacy level of patients with chronic diseases and their quality of life

Individuals with high health literacy can autonomously adopt beneficial lifestyles and behavioral habits to enhance their health. In contrast, those with lower health literacy often experience adverse health outcomes, including reduced disease management capabilities, increased mortality rates, and higher hospitalization rates compared to their counterparts with higher health literacy (32, 33). Research by Matsuoka et al. indicated that patients with heart failure lacking health literacy-related knowledge demonstrate suboptimal self-care practices and adherence to health behaviors, placing them at elevated risk for recurrent hospitalizations due to unstable conditions (34, 35). A meta-analysis conducted by Fabbri et al. (36) further corroborated these findings, revealing that patients with heart failure who have low health literacy have a 1.19 times higher risk of mortality and a 1.17 times higher risk of hospitalization compared to those with higher health literacy. Moreover, Marciano et al.’s study (33) on the health literacy of patients with diabetes highlighted the pivotal role of health literacy in enhancing patients’ understanding of diabetes-related knowledge. Compared to their counterparts lacking health literacy, patients with adequate health literacy demonstrate better blood sugar control, reduced risk of complications, and improved prognosis. This improvement May stem from their enhanced comprehension of medical information and heightened motivation to engage in disease management actively.

Patients with higher health literacy will have enhanced self-discipline and be more active in self-management (37). Therefore, when in contact with medical staff, it is easier to digest and absorb this knowledge after receiving appropriate health guidance so that you can adequately self-care and care for some adverse symptoms. Relevant studies have shown that patients with NCDs who possess higher health literacy levels tend to experience lower levels of anxiety, depression, and other negative emotions. Conversely, there is a positive association between higher health literacy and improved QoL for these patients (38, 39). Simultaneously, individuals with adequate health literacy are better equipped to utilize the available social support network and access material and psychological assistance. This enables them to cope with their illness more effectively. Therefore, in treating and managing patients with NCDs, healthcare providers should understand the relevant factors that affect patients’ health literacy and carry out targeted intervention measures based on these factors to continuously improve their health literacy.

### 4.2 The relationship between self-efficacy and quality of life in patients with chronic diseases

Self-efficacy is a pivotal determinant in the onset and progression of diseases, as it directs individuals toward adopting healthy behaviors. It significantly influences patients’ emotional well-being, psychosocial adjustment, and overall QoL (20, 40). Theoretical frameworks for chronic disease management indicate that self-efficacy predicts health behaviors and coping mechanisms among chronic patients, including their behavioral resilience and stress management capabilities. Individuals with elevated self-efficacy are more likely to sustain and enhance their current health-related life status (41, 42). A study by Wang et al. (43) demonstrated a positive correlation between the self-efficacy of old adult with arteriosclerotic occlusive disease and their QoL, particularly in the context of empathetic nursing’s impact on life quality and treatment adherence among the old adult post-cerebral infarction.

The findings of this study underscore the significant influence of self-efficacy on the QoL among patients with chronic illnesses. Elevated self-efficacy in these patients correlates with a higher QoL, and it has been established as a positive predictor of life quality (*p* < 0.05), aligning with domestic and international research. A randomized controlled trial involving patients with chronic kidney disease demonstrated that health interventions can enhance their self-efficacy, improving their self-management capabilities and life quality (44). A cross-sectional survey of 130 Chinese patients undergoing maintenance hemodialysis revealed that those with lower self-efficacy were more susceptible to blood pressure variability and associated complications (45). Studies have consistently indicated that patients with lower self-efficacy scores tend to have a diminished QoL (46). A longitudinal study of patients with ischemic stroke in Germany showed that diminished self-efficacy is linked to an increased risk of depression (47). Research on chronic obstructive pulmonary disease (COPD) patients has also highlighted that higher self-efficacy is associated with better social functioning and overall health (48). Self-efficacy can shape an individual’s health beliefs, with higher self-efficacy fostering a more resolute problem-solving attitude and more excellent resistance to the negative impact of health-related challenges. Furthermore, it can mitigate the negative emotions from physical discomfort, enhancing an individual’s agency and motivation to adopt healthier lifestyle practices.

### 4.3 The simple mediating role of self-efficacy between health literacy and quality of life in patients with chronic diseases

This study delved into the potential mediating or moderating roles of self-efficacy in the relationship between health literacy and the QoL in patients with NCDs. The findings indicate that self-efficacy mediates this relationship, accounting for 14.5 and 14.8% of the total effect on health literacy and QoL, respectively.

In a study by Lee et al. (49) concerning the determinants of life quality in kidney disease patients, self-efficacy emerged as a mediator in the connection between mental health and life quality. This mediating role of self-efficacy is not merely theoretical; interventions aimed at enhancing self-efficacy have been demonstrated to improve the overall QoL for patients. The significance of this mediating role was further investigated in the context of patients with tuberculosis in Tibet, China, where examining the relationship between health literacy and QoL underscored the importance of self-efficacy and self-management (50). Kim et al. (51) conducted a randomized clinical trial focusing on health literacy in patients with type 2 diabetes, revealing that self-efficacy is a critical mediator between health literacy and glycemic control and QoL. Self-efficacy is pivotal in transforming knowledge into action, as it can stimulate and sustain an individual’s motivation and capacity to engage in healthy behaviors (52). It is recognized that an increase in knowledge does not automatically result in behavioral changes, and traditional health education methods need to be revised for the evolving informational demands of contemporary society. Thus, health promotion must convey knowledge and leverage psychosocial factors, such as self-efficacy, to encourage behavioral shifts toward healthier lifestyles (53, 54). Health literacy indirectly influences the self-efficacy of patients with NCDs, enhancing their confidence and self-management skills (55, 56), which fosters their ability to self-manage their conditions. Studies have demonstrated that patients with greater self-efficacy have higher aspirations and demands for recovery and exhibit better adherence to health management and treatment protocols (57). Moreover, these patients are more likely to confront their illness with a positive outlook and effectively manage negative emotions. Consequently, healthcare professionals should assess patients’ understanding of their current health management and rehabilitation strategies, aiming to enhance their QoL by bolstering self-efficacy and activating their latent coping and problem-solving skills.

### 4.4 Advantages and disadvantages

The strengths of this study lie in its comprehensive scope and a representative sample, which bolsters the credibility of the findings. Utilizing the four-way decomposition method, we have thoroughly investigated the interplay among self-efficacy, health literacy, and the QoL in patients with NCDs, along with their underlying mechanisms. This approach offers a novel perspective for designing future interventions to enhance the life quality of individuals with chronic conditions.

However, the study is subject to limitations. Initially, dependence on self-reported data from participants May introduce measurement bias. Additionally, the study’s cross-sectional design hinders the establishment of causality between variables. Thirdly, the study population in this study was only a sample of Chinese adults, and there was no research population from other countries for verification.

In future research, it is imperative to employ longitudinal or controlled study designs to investigate the temporal dynamics linking health literacy, self-efficacy, and quality of life among patients with NCDs. Moreover, data collection should encompass a range of methods, including qualitative interviews and mixed-methods studies, to provide a comprehensive and nuanced dataset. Given the heterogeneity of populations, influenced by their unique geographical contexts and cultural practices, the quality of life for patients with NCDs is expected to differ across regions. Consequently, there is a need for in-depth, region-specific studies to better understand the particular challenges and needs of these patients.

## 5 Conclusion

The study’s findings demonstrate a positive correlation between self-efficacy and health literacy, the QoL among patients with NCDs. Additionally, self-efficacy indirectly influences the relationship between health literacy and QoL in these patients. Implementing targeted interventions to boost self-efficacy and health literacy is likely to be vital for enhancing the QoL of individuals with NCDs. Moreover, future research should continually assess the post-intervention impacts and interactions of self-efficacy and health literacy on QoL in this patient population.

## Data Availability

The original contributions presented in the study are included in the article/supplementary material, further inquiries can be directed to the corresponding author.
